# Essential oils as growth-promoting additives on performance, nutrient digestibility, cecal microbes, and serum metabolites of broiler chickens: a meta-analysis

**DOI:** 10.5713/ab.20.0668

**Published:** 2020-12-13

**Authors:** Agung Irawan, Cecep Hidayat, Anuraga Jayanegara, Adi Ratriyanto

**Affiliations:** 1 Vocational Program in Animal Husbandry, Vocational School, Universitas Sebelas Maret, Surakarta 57126, Indonesia; 2 Animal Feed and Nutrition Modelling Research Group (AFENUE), Department of Nutrition and Feed Technology, Faculty of Animal Science, IPB University, Bogor 16680, Indonesia; 3 Indonesian Research Institute for Animal Production, Ciawi Bogor 16720, Indonesia; 4 Department of Nutrition and Feed Technology, Faculty of Animal Science, IPB University, Bogor 16680, Indonesia; 5 Department of Animal Science, Faculty of Agriculture, Universitas Sebelas Maret, Surakarta 57126, Indonesia

**Keywords:** Antibiotics, Broiler, Essential Oils, Growth Promoter, Meta-analysis, Production Performance

## Abstract

**Objective:**

The purpose of this meta-analysis was to evaluate the effect of dietary essential oils (EOs) on productive performance, nutrient digestibility, and serum metabolite profiles of broiler chickens and to compare their effectiveness as growth-promoting additives against antibiotics.

**Methods:**

Peer-reviewed articles were retrieved from Web of Science, Science Direct, PubMed, and Google scholar and selected based on pre-determined criteria. A total of 41 articles containing 55 experiments with 163 treatment units were eligible for analyses. Data were subjected to a meta-analysis based on mixed model methodology considering the doses of EOs as fixed effects and the different studies as random effects.

**Results:**

Results showed a linear increase (p<0.001) on body weight gain (BWG) where Antibiotics (FCR) and average daily feed intake decreased (p<0.001) linearly with an increasing dose of EOs. Positive effects were observed on the increased (p<0.01) digestibility of dry matter, crude protein, ether extract, and cecal Lactobacillus while *Escherichia coli* (*E. coli*) population in the cecum decreased (p<0.001) linearly. There was a quadratic effect on the weight of gizzard (p<0.01), spleen (p<0.05), bursa of fabricius (p<0.001), and liver (p< 0.10) while carcass, abdominal fat, and pancreas increased (p<0.01) linearly. The dose of EOs linearly increased high density lipoprotein, glucose, protein, and globulin concentrations (p<0.01). In comparison to control and antibiotics, all type of EOs significantly reduced (p<0.001) FCR and tended to increase (p<0.1) BWG and final body weight. Cinnamaldehyde-compound was the only EOs type showing a tendency to increase (p<0.1) carcass weight, albumin, and protein of serum metabolites while this EOs together with EOs-Blend 1 decreased (p<0.01) *E. coli* population. Low density lipoprotein concentration decreased (p<0.05) with antibiotics and carvacrol-based compound when compared to the control group.

**Conclusion:**

This evidence confirms that EOs are suitable to be used as growth promoters and their economical benefit appears to be promising.

## INTRODUCTION

Global pressure to replace the use of antibiotics as growth promoters (AGPs) with safe feed additives in the broiler industry has led researchers to conduct a massive exploration in utilizing natural substance-based additives. It is primarily due to the AGPs restricted use by the European Union since 2006 concerning antimicrobial resistance, human health, and sustainability [1]. Essential oils (EOs), volatile properties derived from plant materials by mainly steam distillation method, are among promising growth enhancers in broiler chickens that received a growing interest among scholars and industrial stakeholders. EOs are formed by dozens of complex mixture components [2] that can be classified into a group of terpenoids (menthol, linalool, geraniol, borneol, α-terpineol) and a group of low molecular weight aliphatic hydrocarbons (thymol, carvacrol, eugenol, cinnamaldehyde). The advantageous effects of EOs are associated with their role on many metabolic pathways, including on lipid metabolism, stimulate digestive enzyme secretion and activity, act as antimicrobial, and enhance gut integrity of chickens [3,4] leading to improve broiler performance in general.

However, inconsistent results among past and present studies are identified to be conflicting. For instance, there were positive effects of EOs on broiler performance as indicated with improving body weight gain (BWG) [5,6], feed conversion ratio (FCR) [7], enzyme secretion [8], and nutrients digestibility [9–11]. In addition, previous report also revealed that EOs supplementation effectively replaced the use of AGP [12]. On the other hand, there were contrary reports where several authors suggested no effect of dietary EOs on broiler productive performance [13,14] or even had lower weight gain compared to those not receiving AGP or EOs [15]. Major explanations from these contradictory findings have been attributed to the different of EOs active components used in the individual study, their natural origin such as plant source, plant part, geographical condition, and also environmental and physiological factors of the animal used [3,11]. Indeed, the mode of action from specific EOs molecules is limited to elucidate different results from a number of studies. The magnitude of the biological effect of EOs varies depending on the complex chemical structure of EOs.

Comprehensive reviews describing the main effect and mode of action of EOs on poultry have been provided [3,4, 17]. However, to our knowledge, the effects of EOs on broiler chickens have not been summarized quantitatively to date. Data from available empirical studies can be integrated and quantified using a robust statistical method, i.e., the meta-analysis because the method considers heterogeneity among individual studies which increases the power to calculate the treatment effect [18,19]. Therefore, this study aimed to investigate the efficacy of the application of EOs as alternative growth-promoting additives in the diet on the productive performance of broiler chickens.

## MATERIALS AND METHODS

### Database development and inclusion criteria

A database was developed based on scientific publications available online at several search engines such as Web of Science, Science Direct, and PubMed. Keywords used in this study were “broiler performance” and “essential oil” or “phytogenic”. To specify the result of the browsing process, we used several filters available on the website such as type of article, range of year of publication, and journal name. Journal name filter was used to exclude irrelevant journals that appeared during searching the database such as journal related to aquaculture and food science. All relevant titles of publication from the respective websites were further imported into the reference manager for selection purposes.

In total, 124 published articles were identified to match the purpose of this study based on the title of the article. These data were further assessed with qualification criteria developed according to the guidelines of Preferred Reporting Items for Systematic Reviews and Meta-Analyses (PRISMA) to ensure the quality of the meta-analysis [20]. Assessment criteria were, i) research articles published between 2006 to 2019 from a peer-reviewed journal, ii) *in vivo* trials containing control group and EOs treatment group, iii) articles not containing antibiotics used as a growth promoter in the control or EOs group except for the AGP used as a positive control, iv) articles reporting experimental periods, and at least performance parameters of final body weight (BW) or weight gain and feed intake or FCR, and v) articles reporting the EOs sources or major type of EOs and supplementation dose.

**Figure 1:**
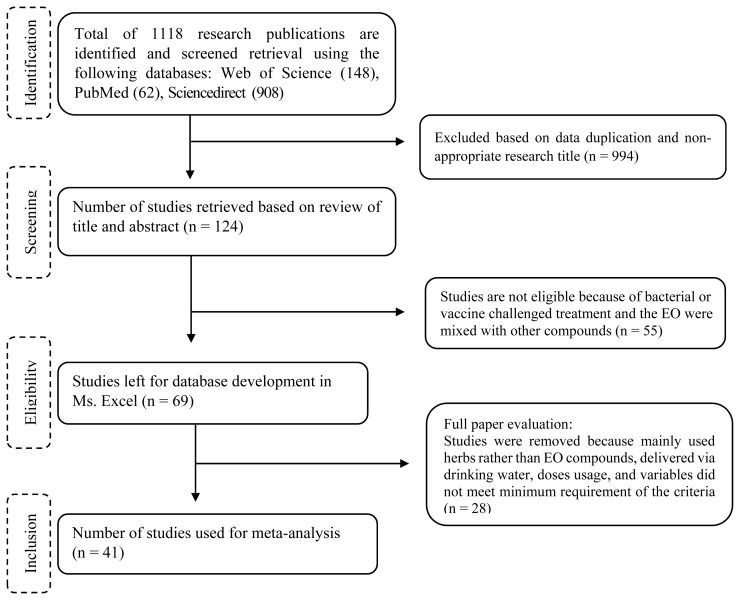
Flow charts of publications utilized for the meta-analysis.

In addition, studies conducted before 2006 were excluded because it was the period in which the use of AGP in feed was permitted thereby difficult to measure the only effect of EOs inclusion. The EOs may also have been supplemented through feed or drinking water. In this study, only those administered in feed were integrated into the database. This meta-analysis also ruled out the bacterial challenged studies because they were not the focus of the current study. Because other substances such as organic acids can potentially interfere with the effect of EOs, studies that used organic acid products containing EOs compounds were excluded. Additionally, EOs treatments with ≥1,000 mg/kg were disregarded because the number of these categories was small (n total of this category = 9 observations). Finally, 41 studies comprising 55 experiments and 163 treatment units were eligible and therefore used for the analysis. Details for the selection process of database development are provided in Figure 1 while the summary of the studies is presented in Table 1.

### Data extraction

The information about the journal, authors, year of study, broiler used (including strain and sex), diet type, length of study, treatment, type and composition of EOs, doses of EOs, nutritional specification of the diet, and the mean value of each parameter contained in the study was recorded in a spreadsheet. The parameters included in the database were productive performance (BW, BWG, feed intake (FI), FCR, average daily feed intake [ADFI]), nutrients digestibility (dry matter [DM], organic matter [OM], crude protein [CP], crude fiber [CF], ether extract [EE]), organ weight, intestinal morphology and cecal microbes, and serum metabolite parameters. Summary of nutrient specification of diets is shown in Table 2 and the descriptive statistics of the response variables is presented in Table 3. To obtain the exact values from graphical data, the relevant figures from the papers were subjected to an online extraction tool of WebPlotDigitizer (https://apps.automeris.io/wpd/). All observed data were transformed into the same measurement units for analysis. The sample size for each parameter was calculated. Several parameters such as mortality, OM and CF digestibility, immunoglobulin M, immunoglobulin Y, and several blood metabolites were not eligible to be included because they had relatively small sample sizes (n<10).

### Description of the database

This study included 16,221 broiler chickens averaging 395 (±87.7) birds per study. The strain used was dominated by Ross 308 which accounted for 73% while Cobb 500 was 17% and the other 10% were Arbor Across and Hubbard. All studies described broiler chickens’ sex used (of which 68% were male, 17% mixed, 10% unsex, and the rest was female). As much as 70.7% of all studies used maize – soybean meal based diet. The average duration of the evaluated study was 36 d, the maximum length was 46 d, and the minimum was 9 d. In 90.2% of the experiments, birds received starter diet, while grower and finisher diets were offered to 54.6% and 58.9% of the experiments, respectively. Nutrient specifications of all experiments were summarized for metabolizable energy (ME, kcal/kg), CP (%), and total lysine (%). As given in Table 2, the average of ME, CP, EE, and lysine of the diet provided to the starter, grower, and finisher phases were appropriate with the nutrient requirement of broiler chickens according to NRC [21].

The EOs are a complex mixture of a variety of bioactive compounds. In application, it is largely administered with one to three major components and some trace bioactive compounds, thus the term of EOs is used onward regardless of their specific composition. The EOs inclusion ranged from 0 (control) to 750 mg/kg and averaged at 112 (±149) mg/kg. The EOs included in the database were given in specific major component of EOs (thymol, carvacrol, cinnamaldehyde), a blend of EOs including as commercial EOs (thymol, carvacrol, eugenol, piperine, cinnamaldehyde, anethole, menthol, y-terpinene, limonene, α-turmerone, p-cymene, camphor, α-pipene, d-limonene, linalool), or given as EOs extracted from specific plant sources (oregano EOs, thyme EOs, mint EOs, rosemary EOs, star anise EOs, cinnamon EOs, basil EOs, caraway EOs, laurel EOs, lemon EOs, sage EOs, tea EOs, turmeric EOs, clove EOs). Antibiotics used as a positive control in the studies were salinomycin, zinc bacitracin, avilamycin, chlortetracycline, virginiamycin, and commercial anticoccidial (Cygro).

### Statistical analysis

Data tabulated in the database were subjected to a mixed model analysis according to St-Pierre [18]. The SAS software was employed to analyze the data following the PROC MIXED procedure (SAS Studio 3.8, University Edition, 2018). The EOs were considered as fixed effects whereas the different studies were taken into account as random effects. The model applied was:

$$Y_{ij}=B_{0}+B_{1}X_{ij}+B_{2}{X_{ij}}^{2}+s_{i}+b_{i}X_{ij}+e_{ij}$$

where *Y_ij_* = the expected output for dependent variable *Y* at level *j* from the variable *X* as a continuous variable in the study *i*, *B*_0_ = overall intercept across experiments (fixed effect), *B*_1_ = linear regression coefficient of *Y* on *X* (fixed effect), *B*_2_ = quadratic regression coefficient of *Y* on *X* (fixed effect), *X*_ij_ = value of the continuous predictor variable *X* (doses of EOs administration), *s*_i_ = random effect of experiment *i*, *b_i_* = random effect of experiment *i* on the regression coefficient of *Y* on *X* in experiment *i* and *e_ij_* = the residual error. In the statement CLASS, the “study” variable was declared. Data were weighted by the number of replicates in each study. Additionally, an unstructured variance – covariance matrix (type = un) was performed at the random effect part of the model to avoid a positive correlation between intercepts and slopes [18]. Regarding the continuous predictor, p-values for intercepts and slopes, Akaike’s information criterion, and root mean square error were used for model statistics [22,23]. Meanwhile, to test the effectiveness of type of EOs, we categorized the EOs according to their major compound and antimicrobial activity following the classification and justification from the evidence of previous reports [3,24]. Here, the EOs groups possible to compare were thymol-based compound (thy-BC), carvacrol-based compound (car-BC), cinnamaldehyde-based compound (cin-BC), and menthol-based compound (men-BC). Treatments containing one or more combinations of these compounds except for menthol were grouped as EOs-Blend 1 (EOB 1) and the other containing multiple compound of terpenoids (linalool, geraniol, thujanol, borneol, menthol, citronellol, terpineol) was grouped as EOs-Blend 2 (EOB 2). We did not group a single compound from terpenoids except for menthol because the sample size was too small. As a result, there were eight treatment groups consisting of six EOs categories as moderating variables, AGP group, and control group. The categorical analysis was performed according to the following model:

$$Y_{ij}=\mu +S_{i}+\tau_{j}+S\tau_{ij}+e_{ij}$$

where *Y_ij_* = the expected output for dependent variable *Y*, *μ* = overall mean, *S_i_* = random effect of *i* study, *τ_j_* = fixed effect of the *j* level, *Sτ_ij_* = random interaction between *i* study and the *j* level, and *e_ij_* = residual error. A significant effect was declared at p<0.05 or there is a tendency when the p-value was between 0.05 and 0.10. Comparison among the experimental group was conducted with least square means and adjusted with Tukey’s test.

## RESULTS

### The dose of EOs administration on productive performance, nutrient digestibility, cecal microbes, and serum metabolites of broiler chickens

The regression equations between the dose of EOs and broiler productive performance, nutrient digestibility, intestinal profile, and serum metabolites are presented in Table 4 and 5. The productive parameters (BWG and final BW) represented a linear increase where ADFI and FCR showed a linear decrease (p<0.001) with increasing EOs doses except for feed intake that showed a quadratic model (p<0.001). Similarly, positive effects of EOs inclusion were also detected on increasing apparent DM, CP, and EE digestibility with a linear pattern (p<0.001) which may be a factor promoting the increase in BWG. Administration of EOs also increased carcass and gizzard and decreased abdominal fat percentage relative to BW (p<0.01) and some of the lymphoid organ weight such as the spleen, bursa fabricius (BF), and pancreas although the pattern of spleen and BF followed quadratic relationship (p<0.05). To a lesser extent, liver tended to show a quadratic pattern (p = 0.054).

Another beneficial effect of supplementing EOs in the broiler diet was shown by linearly increasing (p<0.001) *Lactobacillus* and suppressing *Escherichia coli* (*E. coli*) population in the cecum. The population of coliform also linearly increased (p = 0.03) although the effect was smaller. Villus height of the broiler intestine increased as the EOs doses increased (p<0.001) but the villus width showed a contrary result (p = 0.007). A different response was observed on crypt depth which was affected quadratically by EOs administration (p<0.05) while the height/depth criterion decreased linearly (p<0.05).

Responses of serum metabolites to dietary EOs were positive, as the EOs linearly reduced low density lipoprotein (LDL) concentration (p<0.01) and concomitantly increased high density lipoprotein (HDL), glucose, protein, and globulin concentrations at a linear pattern (p<0.01). The concentration of triglycerides and cholesterol linearly and quadratically decreased in response to elevating the dose of EOs (p<0.01). There was no effect of EOs inclusion on immunoglobulin metabolite (IgG) as shown in Table 5 (p = 0.169).

### Comparison of the effectiveness of the type of EOs and AGP on broiler performance

The effectiveness of EOs to replace the use of AGP in terms of improving broiler performance was examined and the comparison results are presented in Table 6 and 7. There was marginal evidence on the effect of EOs administration on daily weight gain (BWG) and final BW of broiler chickens whereas EOB 1 increased (p<0.05) the BWG and final BW (p<0.05) by 8.52% and 11.17%, respectively compared to those of Con and AB groups. The FCR significantly decreased (p<0.001) by employing all types of EOs (Thy-BC, Car-BC, Cin-BC, Men-BC, EOB 1, and EOB 2) whereas the Car-BC showed the largest reduction (7.14%) in comparison with the control group. There was no effect (p>0.05) of AB on FCR. The inclusion of EOs in this study did not influence (p = 0.956) ADFI and cumulative FI (p = 0.967). Type of EOs also failed to increase the digestibility of DM, CP, and EE compared with the use of AGP and that did not receive any additive (p>0.10). Cin-BC treatment tended to increase (p = 0.067) carcass weight by 5.67% while Thy-BC group tended to increase (p = 0.071) relative weight of liver (Table 7).

The population of *E. coli* significantly reduced (p<0.01) with Cin-BC and EOB 1 treatments by 8.88% and 7.76%, respectively while the population of Coliform and *Lactobacillus* did not differ among the treatment groups (p>0.10). There was no difference in the intestinal morphology of broiler chickens in response to the administration of AGP or type of EOs (p>0.10). Furthermore, due to relatively large variation of the data of immunoglobulin concentration and serum metabolites parameters, most of these parameters including the concentration of IgG, triglycerides, cholesterol, HDL, and glucose did not change with the application of EOs in comparison to AB and control groups (p>0.10). However, a positive effect was observed on significantly reduced (p<0.05) LDL concentration by 14.55% and 17.86%, respectively using AB and Car-BC when compared to the control group. Globulin concentration increased (p<0.05) by 39.43% with Cin-BC treatment while this treatment also tended to increase (p<0.1) the albumin and protein concentration of the serum metabolite.

## DISCUSSION

Empirical works evaluating the efficacy of EOs bioactive compounds on broiler performance are abundant, which of those have been regularly reviewed [3,4,17]. However, no study so far has attempted to quantitatively summarize the effect of EOs on broiler performance by employing a meta-analysis approach. An increase in final BW and a concomitant reduction in FCR of broiler chickens suggested that EOs inclusion promotes more efficient nutrient utilization. This evidence was largely supported by numerous studies with different in their sources and types of EOs, such as Abdel-Wareth et al [25] who used peppermint and menthol, Nameghi et al [26] who used blend EOs from thyme, peppermint, and eucalyptus, and Hashemipour et al [27] who used thymol + carvacrol. Although the effects of EOs on performance are obviously variable within the plant sources, the beneficial effects are apparently identical among different sources and types, and these are dose-dependent. Altop et al [28] revealed a significant quadratic pattern on BW and FCR when using sweetgum (*Liquidambar orientalis* Mill.) leaves that contained y- Terpinen-4-ol and y-terpinene as major components, with an optimum dose of 80 mg/kg. In regard to feed intake, available literatures suggested that EOs effect can be either stimulate or suppress feed palatability, depending on their level, types, and bird age [28–30].

Inconclusive findings among previous studies underlying the mechanism on how dietary EOs could improve broiler performance are at least partially overcome in this current meta-analysis. Such an effect can be attributed to the increasing nutrient digestibility, gizzard weight (capacity), and villus height as shown in the relationship between the dose of EOs and response variables. Previously, similar results were reported that EOs inclusion improved apparent ileal digestibility of nutrients at 21, 35, and 42 d of age [9]. Little is known about the effect of EOs on enzyme secretion at the early age of broiler because, to our knowledge, there is no study examining nutrient digestibility at the age of less than 14 d in regard to EOs incorporated-diet. Thus, it was speculated that EOs may show a lack of ability to promote enzyme secretion in the pre-starter or starter phase of broiler because in this period, indigenous enzyme secretion is very low [31]. It has been recognized that increasing nutrient digestibility primarily occurred on adult birds, apparently due to the improvement of small intestine morphology and microbial balance, and possibly because of the stimulating effect on an increase in bile acids and digestive and pancreatic enzymes secretion [30,32].

Furthermore, most studies supported the available hypothesis that the effect on enzyme was more pronounced in adult birds. For instance, there was a linear increase in trypsin, lipase, and protease activities on the 24-d broiler intestine fed with EOs containing-phytogenic [27]. Similarly, higher activity and production of the pancreatic and digestive enzyme were exhibited on birds received thymol [32] or oregano EOs [30] while Masouri et al [33] also confirmed an increase in digestive juices which has antimicrobial effects by supplementing EOs. In addition to the positive effects aforementioned, it is primarily important to further investigate a factor related to enzyme secretion enhancement by supplementing EOs because few studies also reported absence effect on that [34] which may be due to difference in bioactive compounds, animal age, hygiene, diet type, and also environmental factors [27].

As confirmed in this study, the abundance of *Lactobacillus* increased while the population of *E. coli* decreased, whereas this result is in line with previous studies that reported similar results [26,35]. It has been widely known that EOs bioactive compounds are broad-spectrum antibacterial, antiviral, and antifungal. Using 45 EOs, Chao et al [36] found that all of these compounds effectively inhibited eight genera of bacteria with different degrees of inhibitory effects. Some were more sensitive to either gram-negative or gram-positive bacteria and some others were effective for both types of bacteria, depending on the variation in chemical compositions of the EOs. Interestingly, the relationship between major and trace components of the EOs was also varied among others which can be synergistic, additive, or antagonistic [37,38].

It is generally accepted that there is more than one mode of action regarding their antibacterial role although the specific mechanism is not clear. In regard to their mode of action, EOs bioactivity can be recognized from their multiple benefits such as antimicrobial, antioxidant, immunomodulator, and anti-inflammatory properties [3]. Available explanations suggested that the antimicrobial effect is largely due to the hydrophobicity characteristics which allow the substances to disturb the permeability of the cell wall of bacteria and its mitochondria. EOs components such as carvacrol, eugenol (2-methoxy-4-(2-propenyl)phenol), and thymol, for example, can depolarize the cytoplasmic membrane of gram-negative bacteria by disintegrating the outer membrane of targeted bacteria [39]. Alteration of intestinal flora is one of the important roles of EOs to act as growth-promoting additive. More recently, microbiome study in the cecal part of broiler confirmed an enrichment of phyla Bacteroidetes and genera Alistipes which play at promoting animal growth by supplementing EOs [40].

In addition to the antimicrobial effect, they are also able to stimulate digestive enzyme secretion and activity and increase bile synthesis that can positively increase the digestibility of nutrients. Moreover, many of EOs constituents showed to improve antioxidant status especially EOs extracted from aromatic plants. Their antioxidant activity was also related to the ability of EOs to reduce cell proliferation and act as anti-inflammatory properties. It was reported that EOs could scavenge reactive oxygen species from bacterial phagocytosis process, resulting in the reduction of tissue oxidative damage and inflammation [8].

An increase in villus height in response to dietary EOs was supported by other studies [10,41] but it was not sufficient to elucidate the mode of action. Nevertheless, Hamedi et al [42] reported there was no effect on villus height and crypt depth by incorporating EOs from peppermint and thyme. Because there is limited information on that, several authors suggested that it might be a result of toxins reducing effect in the gastrointestinal tract because of the modulation of microflora [43]. Also, EOs can enhance the mature enterocytes that can improve absorption capacity due to an increase in the villus height and decrease the crypt depth [26].

EOs also exhibited a positive effect on the organ composition of broiler chickens. This is consistent with numerous studies using different EOs sources [9,25,26]. It is well explained that active components of EOs such as carvacrol, thymol, menthol, and p-cymene were involved in the lipids metabolism, particularly on serum cholesterol [44]. It was found that phenol and flavonoids compounds of EOs reduced rate-limiting enzyme involved in cholesterol synthesis, 3 hydroxy-3-methylglutaryl coenzyme A reductase because it is a key enzyme for cholesterol production [45]. As a result, not only less fat accumulation and more carcass portion, but also a positive correlation to the decreased of cholesterol, triglycerides, and LDL concentrations of serum metabolites were produced in this study. At the same time, blood protein, globulin, glucose, and albumin also increased. These were in agreement with several experiments in which dietary EOs extracted from a variety of plants reduced cholesterol levels [46], and increased blood albumin, globulin, and protein [47,48]. EOs supplementation also affected lymphoid organ weight. Previous studies also demonstrated that EOs blend increased BF and thymus [26,49] but in contrast with Rahimi et al [50]. The effect of EOs on immune function of broiler is apparently weaker compared to its effects as a gut modulator, enzyme stimulator, and growth promoter. This is supported that by the fact that the immunoglobulin concentration of IgG was not affected by the EOs administration in this study.

To better understand which type of EOs affected the broiler performance, analysis of moderator variables that may affect the magnitude of EOs was conducted by grouping the EOs based on references evaluating the degree of antimicrobial effects. Accordingly, this study found that EOs blend containing more than two combination of thymol, carvacrol, cinnamaldehyde, and menthol had a greater effect to increase ADG and final BW of broiler chickens than other type of EOs although the effect was not substantial. In agreement with this study, some previous reports showed that combination of thymol and cinnamaldehyde [36,51] or thymol, carvacrol, and cinnamaldehyde [52,53] had greater antimicrobial effect than antibiotics and significantly increased broiler productive performance. In addition, all types of EOs bioactive compounds also significantly reduce FCR compared to the control and antibiotics groups. This result was also in agreement with previous experiments [54,55] who found that the main effect of antibiotics and EOs was more significant on lowering FCR where the effect on BWG and final BW was rather low. Likewise, in this study EOs promoted higher population of Lactobacillus and lower population of *E. coli* than antibiotics which also reasonably increase feed efficiency. This finding suggested that by using EOs, more economic profit seems to be earned as feed conversion efficiency is of the most important indicator in the broiler industry, in the condition when the relative value of EOs products are similar with AGP. Accordingly, investigations on the economic aspect of various conditions are demanding.

## CONCLUSION

The present meta-analysis confirms that there are positive effects on broiler productive performance as the inclusion levels of EOs increase, regardless of type of components used. Across all studies, the growth-promoting effect of EOs is strongly related to their strategic role in many metabolic pathways as antimicrobial, antioxidant, and anti-inflammatory agents resulting in an increase in nutrient digestibility, carcass percentage, gut integrity, and metabolites profile. Most of the parameters outcome show linear patterns, indicating doses of EOs given are effective to facilitate better growth performance of broiler chickens. This study also demonstrated that in comparison to antibiotics, type of EOs have variable results for BWG, final BW, and FCR. Among EOs bioactive compounds, EO-Blend of thymol, carvacrol, and cinnamaldehyde showed higher efficacy in increasing productive performance and feed efficiency. These points confirm that EOs are suitable to be used as growth promoters and their economical benefit may be promising.

## Figures and Tables

**Table 1:** Description of the studies included in the database

Authors	Birds (n)	Period (d)	Essential oils	Dose (mg/kg)
Loh et al [56]	96	0–21	Blend (thy, ane)	0–150
Ciftci et al [57]	240	5–42	Cinnamon EOs	0–500
Isabel and Santos [58]	660	0–46	Blend (clove & cinnamon EOs)	0–100
Malayoğlu et al [30]	450	0–21	oregano EOs	0–500
Tiihonen et al [51]	720	0–42	Blend (thy, cin)	0–20
Amad et al [9]	528	0–42	Blend (thy, ane)	0–750
Amerah et al [59]	192	0–35	Blend (cin, thy)	0–100
Alp et al [60]	1200	0–42	Oregano EOs	0–300
Bravo et al [61]	240	0–21	Blend (car, cin)	0–100
Bozkurt et al [62]	1248	0–42	Blend (car, 1,8-cin, cam, thy)	0–48
Engberg et al [63]	640	0–35	Artemisia annua oil EOs	0–500
Mueller et al [64]	240	0–35	Blend (turmeric, oregano, thyme, rosemary EOs)	0–150
Hashemipour et al [27]	240	0–42	Blend (thy, car)	0–200
Sarica and Corduk [65]	135	0–21	Oregano EOs, pepper EOs	0–250
Betancourt et al [66]	750	0–42	Oregano EOs	0–200
Habibi et al [67]	168	0–49	Ginger root EOs (zin, β-ses, sab, ar-cur, β-bis)	0–150
Humer et al [68]	432	0–35	Blend (thy, ane)	0–150
Khattak et al [5]	960	0–42	Blend (EOs from basil, caraway, laurel, lemon, oregano, sage, tea, and thyme)	0–60
Ghazi et al [69]	120	0–42	Oregano EOs	0–250
Pirgozliev et al [70]	310	0–21	Blend (cin, car)	0–100
Aristimunha et al [12]	930	0–42	Blend (thy, eug, pip)	0–300
Hafeez et al [71]	600	0–42	Blend (men, ane)	0–100
Basmacioğlu-Malayoğlu et al [10]	240	0–42	Blend (car, cum, eug)	0–300
Kim et al [6]	840	0–35	Blend (thy, ane)	0–150
Ding et al [72]	192	0–42	Star anise EOs (ane)	0–200
Masouri et al [33]	288	0–42	Khuzistanica EOs (car)	0–500
Mohiti-Asli and Ghanaatparast-Rashti [73]	200	0–42	Blend (oregano, anise and citrus peel)	0–500
Paraskeuas et al [74]	150	0–42	Blend (men, ane, eug)	0–150
Ri et al [75]	180	0–42	Oregano EOs	0–150
Altop et al [28]	375	0–42	Sweet gum EOs	0–162.2
Giannenas et al [76]	320	0–42	Blend (β-car, men)	0–500
Hosseini and Meimandipour [77]	150	0–42	Thyme EOs	0–80
Mohiti-Asli and Ghanaatparast-Rashti [78]	200	0–42	Blend (oregano, anise and citrus peel) and oregano	0–500
Pirgozliev et al [7]	256	0–21	Blend (cin, car, cap)	0–100
Park and Kim [38]	360	0–42	Blend (thy, eug, pip)	0–300
Reis et al [52]	240	0–42	Blend (car, thy, cin)	0–500
Abdel-Wareth et al [25]	384	0–35	peppermint EOs and men	0–222
Attia et al [11]	216	0–36	Blend (cin, thy)	0–150
Galli et al [53]	135	0–44	Blend (car, thy, cin)	0–100
Nouri [79]	300	0–14	mint EOs, thyme EOs, cinnamon EOs	0–55
Placha et al [80]	96	0–28	Thyme EOs	0–100

EOs, essential oils; thy, thymol; ane, anethole; car, carvacrol; cin, cinnamaldehyde; 1,8-cin, 1,8-cineole; zin, zingiberene; β-ses, β-sesquiphellandrene; sab, sabinene; ar-cur, ar-curcumene; β-bis, β-bisabolene, eug, eugenol; pip, piperine; men, menthol; cum, cuminaldehyde; β-car, β-caryophillene; cap, capsicum oleoresin.

**Table 2:** Descriptive statistics of nutrient specifications used in meta-analysis

Parameters	Unit	n	Mean	SD	Min	Max
Nutrient specification						
Starter						
ME	Kcal/kg	139	3,017	99.9	2,796	3,300
Crude protein	%	147	22.08	1.20	18.77	27.68
Ether extract	%	70	6.32	0.16	3.60	9.81
Lysine	%	117	1.27	0.108	0.97	1.45
Grower						
ME	Kcal/kg	81	3,089	77.3	2,970	3,300
Crude protein	%	89	20.74	1.27	19.00	26.16
Ether extract	%	53	7.19	0.14	5.50	9.10
Lysine	%	61	1.16	0.099	0.97	1.39
Finisher						
ME	Kcal/kg	96	3,136	90.1	3,014	3,300
Crude protein	%	96	19.46	1.45	17.00	24.64
Ether extract	%	41	7.55	0.12	6.50	9.50
Lysine	%	76	1.10	0.10	0.90	1.33

SD, standard deviation; Max, maximum; Min, minimum; ME, metabolizable energy.

**Table 3:** Descriptive statistics of the variables used in meta-analysis

Parameters	Unit	n	Mean	SD	Min	Max
Production performance						
Age	d	163	36.0	9.4	14.0	46.0
Body weight	g	152	2,095	731.6	525.1	3,436
Feed intake	g/period	149	3,525	1,334.5	757.6	5,735
Feed conversion ratio	g feed/g gain	149	1.69	0.21	0.71	2.14
Body weight gain	g/bird/d	152	56.48	13.73	25.01	83.90
Average daily feed intake	g/d	149	95.80	25.31	36.07	152.0
Nutrient digestibility						
Dry matter	%	23	71.20	6.56	56.20	79.01
Crude protein	%	30	72.45	10.51	45.30	86.50
Ether extract	%	20	85.28	6.66	73.30	94.80
Relative organ weight						
Carcass	% BW	34	71.37	0.922	59.48	78.85
Breast meat	% BW	10	28.06	13.86	14.81	45.22
Abdominal fat	% BW	23	1.76	0.62	0.66	2.83
Gizzard	% BW	26	1.98	0.190	0.63	3.40
Lymphoid organ weight						
Liver	% BW	57	2.43	0.088	1.73	5.25
Spleen	% BW	44	0.20	0.35	0.08	1.79
Bursa of Fabricius	% BW	31	0.19	0.03	0.10	0.27
Pancreas	% BW	38	0.45	0.50	0.20	1.75
Cecal microbes						
*Escherichia coli*	Log CFU/g	26	7.03	1.20	5.11	9.56
Lactobacillus	Log CFU/g	30	7.33	1.91	1.97	9.81
Coliform	Log CFU/g	21	7.19	0.97	5.49	8.40
Intestinal morphology						
Villus height	μm	29	858.0	465.1	286.0	1752
Villus width	μm	14	148.7	87.7	80.5	404.4
Crypth depth	μm	29	154.9	15.92	60.0	404.0
Height/depth	μm	12	5.35	2.44	2.67	9.10
Serum metabolites						
IgG	μg/mL	13	13.62	11.75	4.03	39.41
Triglycerides	mg/dL	39	92.02	46.81	28.60	178.0
Cholesterol	mg/dL	44	138.80	80.82	42.00	443.8
HDL	mg/dL	18	78.42	25.21	50.20	139.9
LDL	mg/dL	15	79.35	77.80	33.80	273.0
Glucose	mg/dL	31	249.9	5.32	189.3	320.2
Albumin	mg/dL	15	2.16	0.81	0.92	3.70
Protein	mg/dL	25	4.35	2.03	1.36	7.60
Globulin	mg/dL	12	2.19	0.633	1.70	3.80

SD, standard deviation; Max, maximum; Min, minimum; IgG, immunoglobulin G; HDL, high density lipoprotein; LDL, low density lipoprotein.

**Table 4:** Regression equations on the effect of essential oils dose (in mg/kg of diet) on production performance, nutrient digestibility, and organ weight of broiler chickens

Response variables	n	Model	Variable estimates				Model estimates			
	
			Intercept	SE_Intercept_	Slope	SE_Slope_	p-value	RMSE	AIC^1)^	BIC^2)^
Production performance										
Body weight (g)	152	L	2,065	110.5	0.1668	0.1791	<0.0001	267.79	2,267	2,283
Feed intake (g/period)	149	Q	3,482	207.6	−0.2197	0.3095	<0.0001	445.54	2,392	2,396
					−0.0005	0.0014	<0.0001			
Feed conversion ratio	149	L	1.67	0.031	−0.0002	0.00004	<0.0001	0.06	−212	−208
Body weight gain (g/bird·d)	152	L	55.7	2.03	0.004	0.004	<0.0001	5.24	1,082	1,104
ADFI (g/bird/d)	149	L	94.5	3.90	−0.006	0.006	<0.0001	8.78	1,225	1,228
Nutrient digestibility (%)										
Dry matter	23	L	71.31	2.108	0.0019	0.0049	<0.0001	2.83	141	145
Crude protein	30	L	73.71	2.505	0.0048	0.0068	<0.0001	5.58	215	218
Ether extract	20	L	85.92	2.369	0.0058	0.0051	<0.0001	3.83	129	133
Relative organ weight (% BW)										
Carcass	34	L	60.67	2.078	0.004	0.003	<0.0001	1.45	167	170
Breast meat	10	L	23.51	7.651	0.006	0.002	0.054	0.68	55	58.6
Abdominal fat	23	L	1.88	0.203	−0.0001	0.0003	<0.0001	0.17	31	34.4
Gizzard	26	Q	3.00	0.711	0.000573	0.0012	0.004	0.31	91	94.2
					−2.24E-6	3.177E-6	<0.01			
Lymphoid organ weight (% BW)										
Liver	57	Q	5.52	2.622	0.001048	0.001163	0.067	0.51	235	239
					−4.83E-6	2.071E-6	0.054			
Spleen	44	Q	0.25	0.108	−0.00001	0.000035	0.057	0.01	−98	−94.4
					6.918E-8	0.00001	0.042			
Bursa of Fabricius	31	Q	0.19	0.012	−2.73E-6	0.000094	<0.0001	0.02	−73	−69.3
					−2.27E-9	0.00001	<0.0001			
Pancreas	38	L	0.58	0.156	0.0001	0.00004	0.006	0.03	−70	−66

SE, standard error; RMSE, root mean square error; AIC, akaike information criterion; BIC, bayesian information criterion; L, linear; Q, quadratic; ADFI, average daily feed intake; BW, body weight.

1) AIC is an estimator of the relative quality of statistical models for a given set of data (smaller is better).

2) BIC is an estimator of a probability of a model being true (smaller is better).

**Table 5:** Regression equations on the effect of essential oils dose (in mg/kg of diet) on cecal microbes, intestinal morphology, and serum metabolites profile of broiler chickens

Response variables	n	Model	Variable estimates				Model estimates			
	
			Intercept	SE_Intercept_	Slope	SE_Slope_	p-value	RMSE	AIC^1)^	BIC^2)^
Cecal microbes (Log CFU/g)										
*Escherichia coli*	26	L	6.98	0.440	−0.0002	0.0006	<0.0001	0.46	76	79.4
Lactobacillus	30	L	7.10	0.645	0.0010	0.0003	<0.0001	0.26	74	77.8
Coliform	21	L	5.42	1.648	0.0003	0.0006	0.03	0.37	57	60.7
Intestinal morphology (μm)										
Villus height	29	L	920.1	163.03	0.198	0.102	0.0005	88.13	375	379
Villus width	14	L	129.9	19.98	−0.018	0.034	0.0074	15.26	127	131
Crypth depth	29	Q	170.5	30.46	−0.1023	0.09882	0.0006	23.12	313	317
					−0.102	0.099	0.0005			
Height/depth	12	L	5.6	1.25	−0.0002	0.002	0.0211	1.55	63	66.2
Serum metabolites										
IgG (μg/mL)	13	L	10.99	6.107	0.013	0.004	0.169	2.43	83	86.7
Triglycerides (mg/dL)	39	Q	87.68	14.056	0.01704	0.02387	0.0001	7.50	334	338
					−0.00008	0.00005	0.0002			
Cholesterol (mg/dL)	44	Q	131.05	22.050	−0.04666	0.04605	0.0001	14.73	432	436
					0.000209	0.0001	<0.0001			
HDL (mg/dL)	18	L	75.88	13.990	0.019	0.009	0.0056	5.65	139	143
LDL (mg/dL)	15	L	46.16	3.489	−0.022	0.007	0.0009	3.56	97	100
Glucose (mg/dL)	28	L	240.18	11.120	0.050	0.032	<0.0001	14.92	251	254
Albumin (mg/dL)	15	Q	1.62	0.253	0.002053	0.0014	0.0032	0.18	47	50.5
					−5.13E-6	3.068E-6	0.0031			
Protein (mg/dL)	25	L	4.18	0.616	0.001	0.001	0.0011	0.55	76	79.5
Globulin (mg/dL)	12	L	1.93	0.329	0.001	0.001	0.0099	0.68	41	44.9

SE, standard error; RMSE, root mean square error; AIC, akaike information criterion; BIC, bayesian information criterion; CFU, colony forming unit; IgG, Immunoglobulin G; HDL, high density lipoprotein; LDL, low density lipoprotein.

1) AIC is an estimator of the relative quality of statistical models for a given set of data (smaller is better).

2) BIC is an estimator of a probability of a model being true (smaller is better).

**Table 6:** Effect of antibiotics and EOs administration on production performance, nutrient digestibility, and organ weight of broiler chickens

Response variables	Unit	n	Con^1)^	AB^1)^	Type of EOs bioactive compounds^1)^						SEM	p-value

					Thy-BC	Car-BC	Cin-BC	Men-BC	EOB 1	EOB 2		
Production performance												
Body weight	g	152	2,042^b^	2,075^ab^	1,945^c^	2,174^ab^	2,096^ab^	2,120^ab^	2,270^a^	2,082^ab^	59.34	0.080
Feed Intake	g	149	3,476	3,487	3,213	3,440	3,451	3,445	3,718	3,480	109.33	0.304
Feed conversion ratio	g feed/g gain	149	1.68^a^	1.66^ab^	1.62^b^	1.56^c^	1.62^b^	1.60^b^	1.64^b^	1.65^b^	0.02	<0.001
Body weight gain	g/ bird/d	152	55.05^b^	55.71^b^	53.96^c^	57.87^ab^	57.72^ab^	56.73^b^	59.74^a^	56.32^b^	1.11	0.059
Nutrient digestibility												
Dry matter	%	23	70.85	71.79	69.57	-	73.64	66.58	73.6	73.28	1.37	0.472
Crude protein	%	30	72.41	74.46	73.13	-	79.38	79.65	75.51	76.19	1.92	0.635
Ether extract	%	20	84.98	83.86	86.61	-	90.88	87.22	90.07	83.46	1.49	0.329
Relative organ weight												
Carcass	% BW	34	69.36^b^	69.88^b^	71.19^ab^	69.84^b^	73.29^a^	69.69^b^	69.29^b^	67.73^b^	0.91	0.067
Abdominal fat	% BW	23	1.89	2.13	1.96	-	1.81	-	2.01	1.57	0.13	0.173
Lymphoid organ weight												
Liver	% BW	57	2.32^b^	2.2^b^	2.58^a^	2.34^ab^	2.11^b^	2.11^b^	2.09^b^	2.5^ab^	0.09	0.071
Spleen	% BW	44	0.24	0.26	0.24	0.25	0.24	0.25	0.25	0.24	0.05	0.914
Bursa of Fabricius	% BW	31	0.19	0.19	0.21	0.19	0.20	-	0.19	-	0.01	0.178
Pancreas	% BW	38	0.58	0.57	0.62	-	0.59	0.58	0.54	0.62	0.08	0.213

EOs, essential oils; SEM, standard error of means; BW, body weight.

1) Con, control; AB, antibiotics; Thy-BC, thymol-based compound; Car-BC, carvacrol-based compound; Cin-BC; cinnamaldehyde-based compound; Men-BC, menthol-based compound; EOB 1, essential oils blend containing one of more of thymol, carvacrol, and cinnamaldehyde compounds; EOB 2, essential oils blend based on terpenoids group (linalool, geraniol, thujanol, borneol, menthol, citronnillol, terpineol).

a-c Value with different letters differ at p<0.05.

**Table 7:** Effect of antibiotics and EOs administration on cecal microbes, intestinal morphology, and serum metabolites profile of broiler chickens

Response variables	Unit	n	Con^1)^	AB^1)^	Type of EO bioactive compounds^1)^						SEM	p-value

					Thy-BC	Car-BC	Cin-BC	Men-BC	EOB 1	EOB 2		
Cecal microbes												
*Escherichia coli*	Log CFU/g	26	7.09^a^	7.18^a^	6.92^ab^	6.91^ab^	6.46^b^	6.82^ab^	6.54^b^	6.95^ab^	0.24	<0.01
Lactobacillus	Log CFU/g	30	7.08	7.21	7.35	7.16	7.32	7.13	7.34	7.82	0.35	0.501
Coliform	Log CFU/g	21	5.49	5.8	5.32	5.54	-	-	-	5.73	0.21	0.559
Intestinal morphology												
Villus height	um	29	914	960	1,149	966	-	-	929	1143	81.21	0.234
Crypth depth	um	29	171	174	192	166	-	-	155^b^	180	14.13	0.153
Heigh/depth	um	12	5.26	5.59	-	5.85	-	-	6.39	4.16	0.71	0.928
Serum metabolites												
IgG	μg/mL	13	10.98	10.63	20.77	11.11	-	-	13.14	-	3.26	0.202
Tryglicerides	mg/dL	39	87.95	86.2	85.27	75.32	86.64	-	84.77	90.12	7.46	0.573
Cholesterol	mg/dL	44	131.7	128.6	138.6	135.1	129.6	-	135.2	130.3	12.18	0.976
HDL	mg/dL	18	75.02	80.39	83.09	81.62	-	-	-	78.18	5.94	0.434
LDL	mg/dL	15	48.08^a^	41.08^b^	35.79^bc^	39.49^b^	-	-	-	43.9^ab^	1.67	<0.05
Glucose	mg/dL	28	236	249	245	-	241	-	239	255	5.32	0.224
Albumin	mg/dL	15	1.59^b^	1.59^b^	1.43^b^	-	2.05^a^	-	1.53^b^	1.69^b^	0.21	0.061
Protein	mg/dL	25	4.01^b^	3.92^b^	4.05^b^	-	5.36^a^	-	3.97^b^	4.29^b^	0.41	0.059
Globulin	mg/dL	12	1.72^b^	1.77^b^	2.07^b^	-	2.84^a^	-	1.81^b^	-	0.16	<0.05

EOs, essential oils; SEM, standard error of means; CFU, colony forming unit; IgG, immunoglobulin G; HDL, high density lipoprotein; LDL, low density lipoprotein.

1) Con, control; AB, Antibiotics; Thy-BC, thymol-based compound; Car-BC, carvacrol-based compound; Cin-BC; cinnamaldehyde-based compound; Men-BC, menthol-based compound; EOB 1, essential oils blend containing one of more of thymol, carvacrol, and cinnamaldehyde compounds; EOB 2, essential oils blend based on terpenoids group (linalool, geraniol, thujanol, borneol, menthol, citronnillol, terpineol).

a-c Value with different letters differ at p<0.05.
